# [2,2′-(2,6,9,13-Tetra­azatetra­decane-1,14-di­yl)diphenolato]iron(III) iodide

**DOI:** 10.1107/S1600536810018787

**Published:** 2010-05-26

**Authors:** Gervas Assey, Ray J. Butcher, Yilma Gultneh, Teshome Yisgedu

**Affiliations:** a Department of Chemistry, Howard University, 525 College Street NW, Washington, DC 20059, USA

## Abstract

The title Fe^III^ complex, [Fe(C_22_H_32_N_4_O_2_)]I, contains a six-coordinate FeN_4_O_2_ cation in which the ligand is a reduced Schiff base resulting from the NaBH_4_ reduction of the condensation product between salicylaldehyde and 1,5,8,12-tetra­azadodecane. In spite of the increased flexibility of the saturated backbone of the ligand compared to the Schiff base from which it was synthesized, the complex adopts a *cis*-FeN_4_O_2_ conformation for the phenolic O-atom donors, which contrasts with the *trans* conformation adopted by the analogous ClO_4_^−^ salt [Yisgedu *et al.* (2009 ▶). *J. Chem. Crystallogr.* **39**, 315–319]. In addition to extensive N—H⋯I hydrogen bonding between the amine H atoms and the anion there is a weak C—H⋯I inter­action.

## Related literature

For early literature related to hexa­dentate ligands, see: Dwyer & Lions (1947 ▶); Das Sarma & Bailar (1955 ▶). For geometric changes from *cis* to *trans*, see: Bera *et al.* (2005 ▶); Boinnard *et al.* (1994 ▶); Dorbes *et al.* (2005 ▶); Floquet *et al.* (2004 ▶); Hayami *et al.* (1997 ▶); Ito *et al.* (1983 ▶); Maeda *et al.* (1991 ▶); McPartlin *et al.* (1978 ▶); Nishida *et al.* (1987 ▶); Salmon *et al.* (1999 ▶); Sinn *et al.* (1978 ▶). For complexes of reduced Schiff bases, see: Harpstrite *et al.* (2003 ▶). For the analogous ClO_4_^−^ salt, see: Yisgedu *et al.* (2009 ▶). 
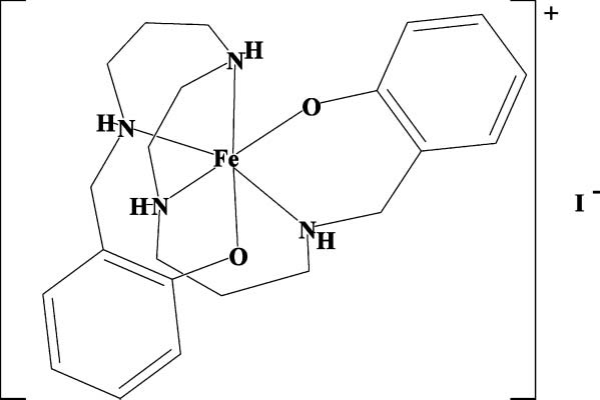


## Experimental

### 

#### Crystal data

[Fe(C_22_H_32_N_4_O_2_)]I
*M* *_r_* = 567.27
Orthorhombic, 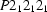

*a* = 9.3958 (1) Å
*b* = 13.0509 (1) Å
*c* = 19.4047 (3) Å
*V* = 2379.48 (5) Å^3^
*Z* = 4
Mo *K*α radiation
μ = 1.96 mm^−1^
*T* = 200 K
0.51 × 0.47 × 0.39 mm

#### Data collection

Oxford Diffraction Gemini R diffractometer
Absorption correction: multi-scan (*CrysAlis RED*; Oxford Diffraction, 2007 ▶) *T*_min_ = 0.428, *T*_max_ = 0.466
43714 measured reflections
9836 independent reflections
7672 reflections with *I* > 2σ(*I*)
*R*_int_ = 0.036

#### Refinement

*R*[*F* ^2^ > 2σ(*F* ^2^)] = 0.033
*wR*(*F* ^2^) = 0.066
*S* = 0.94
9836 reflections
271 parameters
H-atom parameters constrained
Δρ_max_ = 1.32 e Å^−3^
Δρ_min_ = −0.45 e Å^−3^
Absolute structure: Flack (1983 ▶), 4113 Friedel pairs
Flack parameter: −0.018 (11)

### 

Data collection: *CrysAlis CCD* (Oxford Diffraction, 2007 ▶); cell refinement: *CrysAlis RED* (Oxford Diffraction, 2007 ▶); data reduction: *CrysAlis RED*; program(s) used to solve structure: *SHELXS97* (Sheldrick, 2008 ▶); program(s) used to refine structure: *SHELXL97* (Sheldrick, 2008 ▶); molecular graphics: *SHELXTL* (Sheldrick, 2008 ▶); software used to prepare material for publication: *SHELXTL*.

## Supplementary Material

Crystal structure: contains datablocks I, global. DOI: 10.1107/S1600536810018787/zl2278sup1.cif

Structure factors: contains datablocks I. DOI: 10.1107/S1600536810018787/zl2278Isup2.hkl

Additional supplementary materials:  crystallographic information; 3D view; checkCIF report

## Figures and Tables

**Table 1:** Hydrogen-bond geometry (Å, °)

*D*—H⋯*A*	*D*—H	H⋯*A*	*D*⋯*A*	*D*—H⋯*A*
N1*A*—H1*AA*⋯I	0.93	2.77	3.6800 (16)	168
N2*A*—H2*AA*⋯I^i^	0.93	2.96	3.8227 (17)	155
N1*B*—H1*BA*⋯I^i^	0.93	2.80	3.7285 (16)	178
N2*B*—H2*BA*⋯I	0.93	2.81	3.6911 (17)	158
C11*B*—H11*C*⋯I^i^	0.99	3.10	3.942 (2)	144

Symmetry code: (i) 
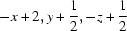
.

**Fig. 1.**
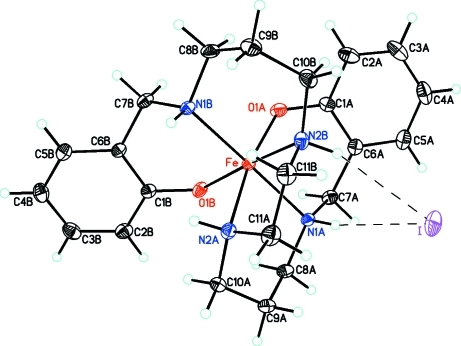
Diagram of C22H32FeIN4O6 showing atom labeling. Thermal ellipsoids are at the 50% probability level.

**Fig. 2.**
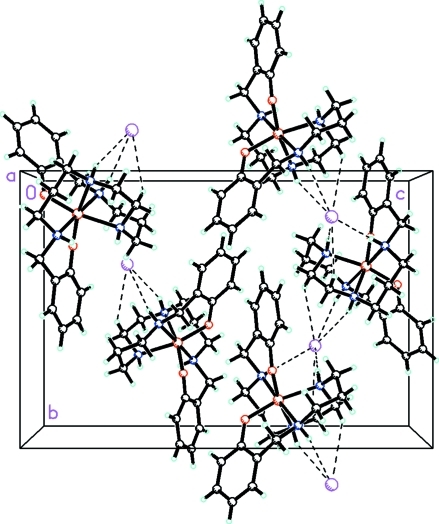
The molecular packing for C22H32FeIN4O6 viewed down the a axis. Hydrogen bonds are shown by dashed lines.

## supplementary crystallographic information

### Comment

Metal complexes of linear hexadentate ligands have fascinated inorganic chemists since their first report in 1947 (Dwyer & Lions, 1947). The first such report of an Fe complex of a linear FeN_4_O_2_ ligand derived from the Schiff base condensation of salicylaldehyde and triethylenetetraamine was in 1955 (Das Sarma & Bailar, 1955). However, this interest lapsed for several years until the discovery that such complexes exhibited spin-crossover magnetic behavior (Sinn *et al.*, 1978). Hexadentate linear FeN_4_O_2_ ligands derived from the Schiff base condensation of salicylaldehyde and linear tetramines can be characterized by the number of linking carbon atoms in the tetramine backbone (from 222 to 333). The structures of Fe complexes of Sal222 (Sinn *et al.*, 1978; Hayami *et al.*, 1997; Floquet *et al.*, 2004; Dorbes *et al.*, 2005; Bera *et al.*, 2005; Nishida *et al.*, 1987; Salmon *et al.*, 1999; McPartlin *et al.*, 1978; Maeda *et al.*, 1991; Boinnard *et al.*, 1994), Sal232 (Hayami *et al.*, 1997), Sal323 (Hayami *et al.*, 1997; Ito, *et al.*, 1983), and Sal333 (Ito, *et al.*, 1983) derivatives have been reported. When chelating to Fe, as the number of carbon atoms in the tetramine backbone increases from 6 to 9, the conformation adopted by the ligand changes from a cis-FeN_4_O_2_ to a trans-FeN_4_O_2_ arrangement for the phenolic O donors. All structurally characterized Fe complexes with Sal222 have adopted the cis-FeN_4_O_2_ conformation while all those with either Sal323 or Sal333 have adopted the trans-FeN_4_O_2_ conformation. For Sal232, both conformations have been observed (Hayami *et al.*, 1997). Further, it has been observed that, in addition to the usual reduction in metal ligand bond distances when going from high spin to low spin, the angles subtended at the Fe center reflect the magnetic properties of the compound (Hayami *et al.*, 1997; Nishida *et al.*, 1987) with low-spin compounds having such angles closer to 90° and 180°.

Despite the interest shown in salicylaldimine complexes with Fe^III^ due to their interesting structural and magnetic properties, there have been very few structures reported on related complexes where the C=N imine groups have been reduced to C–N–H amine groups (Harpstrite *et al.*, 2003; Yisgedu *et al.*, 2009). One of these is the perchlorate analog of the title compound (Harpstrite *et al.*, 2003). As expected, due to increased flexibility of the saturated amine, compared to the more rigid Schiff base, and also the length of the carbon backbone, this compound has adopted a trans-FeN_4_O_2_ conformation. To further characterize such compounds and determine the conformation adopted the structure of an Fe^III^ complex of the iodide salt of reduced Sal323 is reported.

The title compound, [1,12-bis(2-hydroxybenzyl)-1,5,8,12-tetraazadodecane]iron(III) iodide, C_22_H_32_FeIN_4_O_6_, contains a six-coordinate FeN_4_O_2_ cation where the ligand (H_2_L) is the NaBH_4_ reduction product of the Schiff base resulting from the condensation of salicylaldehyde and 1,4,8,12-tetraazadodecane. In marked contrast to the perchlorate salt with the same 323 backbone, the title compound has adopted a cis FeN_4_O_2_ conformation. It is of interest to compare the metrical parameters of both the cis and trans structures with the same central Schiff base core. In the title compound, the Fe—O distances are shorter [1.8898 (13)/1.8999 (14) Å versus 1.9575 (10)/1.9142 (10) Å] while the Fe—N distances are longer [Fe—N average of 2.202 (1) Å versus 2.162 (1) Å]. Thus, even though they adopt different conformations, the bond distances and angles of both the perchlorate and iodide salts are more indicative of a high spin Fe^III^ complex compared to the similar reduced 232 complex (Yisgedu *et al.*, 2009).

In addition to extensive hydrogen bonding between the amine H atoms and the anion there is a weak C—H···I interaction (see table 1).

### Experimental

Synthesis of ligand: The procedure for the synthesis of the ligand 1,12-bis(2-hydroxybenzyl)-1,4,8,12-tetraazaundecane (H_2_L) (Yisgedu *et al.*, 2009) is as follows: A solution of 6.1 g (50 mmol) of salicylaldehyde in 10 ml ethanol was added drop-wise to a solution of 4.0 g (25 mmol) of 1,5,8,12-tetraazadodecane in 15 ml of ethanol. A deep yellow solution was obtained and was stirred for half an hour. To this yellow solution was added a NaBH_4_ solution (3.0 g NaBH_4_, 0.4 g NaOH, and 40 ml H_2_O). The volume of the solution was reduced to 20 ml and extracted with chloroform (3 x 40 ml). The extracts were combined and dried with Na_2_SO_4_. The Na_2_SO_4_ was filtered and the filtrate concentrated to a colorless thick oil (8.1 g, 87%).

Synthesis of [Fe^III^L](ClO_4_): The synthesis of the above complex (Yisgedu *et al.*, 2009) is as follows: To 0.85 g (2 mmol) of H_2_L dissolved in 10.0 ml of methanol was added 0.58 g (1 mmol) of Fe(ClO_4_)_2_.xH_2_O. The solution became violet and red-purple solids precipitated. This was stirred overnight, the solids filtered, washed with methanol and dried to give 1.65 g of red powder. Crystallization was effected by evaporation of a DMF solution of the complex (yield, 0.96 g, 67%).

Synthesis of [C_22_H_32_FeN_4_O_2_]I complex: A solution of 0.05 g (0.088 mmol) of the complex [Fe^III^L]ClO_4_ was mixed with a solution of 10 % w/v aqueous solution of iodine and potassium iodide. 0.095 g of the aqueous solution of iodine/KI mixture in 5 ml methanol was mixed with the complex. The mixture was then stirred at room temperature for 24 hours. The solution was then evaporated, dissolved in DMF and filtered. The filtrate was layered with diethyl ether. After the diffusion process, brownish red crystals suitable for x-ray diffraction were obtained.

### Crystal data

**Table:**

[Fe(C_22_H_32_N_4_O_2_)]I	*F*(000) = 1148
*M**_r_* = 567.27	*D*_x_ = 1.583 Mg m^−^^3^
Orthorhombic, *P*2_1_2_1_2_1_	Mo *K*α radiation, λ = 0.71073 Å
Hall symbol: P 2ac 2ab	Cell parameters from 18399 reflections
*a* = 9.3958 (1) Å	θ = 4.6–34.7°
*b* = 13.0509 (1) Å	µ = 1.96 mm^−^^1^
*c* = 19.4047 (3) Å	*T* = 200 K
*V* = 2379.48 (5) Å^3^	Chunk, dark brown-red
*Z* = 4	0.51 × 0.47 × 0.39 mm

### Data collection

**Table:**

Oxford Diffraction Gemini R diffractometer	9836 independent reflections
Radiation source: fine-focus sealed tube	7672 reflections with *I* > 2σ(*I*)
graphite	*R*_int_ = 0.036
Detector resolution: 10.5081 pixels mm^-1^	θ_max_ = 34.8°, θ_min_ = 4.7°
φ and ω scans	*h* = −14→14
Absorption correction: multi-scan (*CrysAlis RED*; Oxford Diffraction, 2007)	*k* = −20→20
*T*_min_ = 0.428, *T*_max_ = 0.466	*l* = −30→31
43714 measured reflections	

### Refinement

**Table:**

Refinement on *F*^2^	Secondary atom site location: difference Fourier map
Least-squares matrix: full	Hydrogen site location: inferred from neighbouring sites
*R*[*F*^2^ > 2σ(*F*^2^)] = 0.033	H-atom parameters constrained
*wR*(*F*^2^) = 0.066	*w* = 1/[σ^2^(*F*_o_^2^) + (0.0354*P*)^2^] where *P* = (*F*_o_^2^ + 2*F*_c_^2^)/3
*S* = 0.94	(Δ/σ)_max_ = 0.001
9836 reflections	Δρ_max_ = 1.32 e Å^−^^3^
271 parameters	Δρ_min_ = −0.45 e Å^−^^3^
0 restraints	Absolute structure: Flack (1983), 4113 Friedel pairs
Primary atom site location: structure-invariant direct methods	Flack parameter: −0.018 (11)

### Special details

**Table:**

Geometry. All esds (except the esd in the dihedral angle between two l.s. planes) are estimated using the full covariance matrix. The cell esds are taken into account individually in the estimation of esds in distances, angles and torsion angles; correlations between esds in cell parameters are only used when they are defined by crystal symmetry. An approximate (isotropic) treatment of cell esds is used for estimating esds involving l.s. planes.
Refinement. Refinement of *F*^2^ against ALL reflections. The weighted *R*-factor wR and goodness of fit *S* are based on *F*^2^, conventional *R*-factors *R* are based on *F*, with *F* set to zero for negative *F*^2^. The threshold expression of *F*^2^ > σ(*F*^2^) is used only for calculating *R*-factors(gt) etc. and is not relevant to the choice of reflections for refinement. *R*-factors based on *F*^2^ are statistically about twice as large as those based on *F*, and *R*- factors based on ALL data will be even larger.

### Fractional atomic coordinates and isotropic or equivalent isotropic displacement parameters (Å^2^)

**Table:**

	*x*	*y*	*z*	*U*_iso_*/*U*_eq_	
I	0.773383 (17)	−0.160323 (12)	0.264361 (9)	0.04200 (5)	
Fe	0.82534 (3)	0.15269 (2)	0.131418 (12)	0.01814 (5)	
O1A	0.79828 (16)	0.08061 (11)	0.04737 (7)	0.0277 (3)	
O1B	0.71110 (15)	0.26909 (10)	0.11418 (7)	0.0254 (3)	
N1A	0.64230 (16)	0.07176 (12)	0.17734 (8)	0.0203 (3)	
H1AA	0.6763	0.0195	0.2054	0.024*	
N2A	0.86624 (18)	0.20378 (12)	0.23797 (9)	0.0247 (3)	
H2AA	0.9344	0.2551	0.2342	0.030*	
N1B	1.01197 (16)	0.23195 (12)	0.09118 (8)	0.0199 (3)	
H1BA	1.0639	0.2579	0.1281	0.024*	
N2B	0.97740 (18)	0.03588 (13)	0.16943 (9)	0.0250 (3)	
H2BA	0.9218	−0.0207	0.1805	0.030*	
C1A	0.7469 (2)	−0.01336 (15)	0.03747 (10)	0.0243 (4)	
C2A	0.8057 (3)	−0.07758 (19)	−0.01269 (12)	0.0385 (5)	
H2AB	0.8846	−0.0551	−0.0393	0.046*	
C3A	0.7485 (3)	−0.17422 (19)	−0.02343 (14)	0.0450 (6)	
H3AA	0.7890	−0.2178	−0.0574	0.054*	
C4A	0.6331 (3)	−0.20817 (18)	0.01467 (13)	0.0447 (6)	
H4AA	0.5944	−0.2744	0.0068	0.054*	
C5A	0.5753 (3)	−0.14535 (17)	0.06366 (11)	0.0352 (5)	
H5AA	0.4969	−0.1691	0.0902	0.042*	
C6A	0.6286 (2)	−0.04757 (15)	0.07560 (10)	0.0253 (4)	
C7A	0.5530 (2)	0.02586 (16)	0.12218 (10)	0.0250 (4)	
H7AA	0.5130	0.0819	0.0938	0.030*	
H7AB	0.4722	−0.0104	0.1440	0.030*	
C8A	0.5495 (2)	0.14035 (17)	0.21940 (11)	0.0293 (4)	
H8AA	0.4621	0.1025	0.2322	0.035*	
H8AB	0.5206	0.1996	0.1908	0.035*	
C9A	0.6201 (3)	0.17974 (17)	0.28458 (11)	0.0327 (5)	
H9AA	0.5473	0.2158	0.3124	0.039*	
H9AB	0.6530	0.1201	0.3117	0.039*	
C10A	0.7452 (2)	0.25153 (15)	0.27417 (10)	0.0305 (4)	
H10A	0.7129	0.3118	0.2475	0.037*	
H10B	0.7780	0.2762	0.3197	0.037*	
C11A	0.9351 (3)	0.12131 (18)	0.27913 (12)	0.0338 (5)	
H11A	0.8624	0.0716	0.2948	0.041*	
H11B	0.9814	0.1512	0.3203	0.041*	
C1B	0.74548 (19)	0.36819 (14)	0.10896 (9)	0.0226 (4)	
C2B	0.6501 (2)	0.44332 (17)	0.13160 (11)	0.0310 (4)	
H2BB	0.5632	0.4239	0.1529	0.037*	
C3B	0.6827 (3)	0.54589 (17)	0.12298 (12)	0.0352 (5)	
H3BA	0.6161	0.5965	0.1371	0.042*	
C4B	0.8109 (2)	0.57591 (16)	0.09400 (12)	0.0329 (5)	
H4BA	0.8326	0.6466	0.0887	0.040*	
C5B	0.9073 (2)	0.50167 (15)	0.07286 (11)	0.0273 (4)	
H5BA	0.9963	0.5219	0.0540	0.033*	
C6B	0.8749 (2)	0.39720 (14)	0.07890 (10)	0.0216 (4)	
C7B	0.9688 (2)	0.31914 (15)	0.04574 (10)	0.0247 (4)	
H7BA	1.0560	0.3539	0.0291	0.030*	
H7BB	0.9189	0.2910	0.0050	0.030*	
C8B	1.1072 (2)	0.16359 (18)	0.04945 (10)	0.0285 (4)	
H8BA	1.1804	0.2063	0.0266	0.034*	
H8BB	1.0498	0.1306	0.0129	0.034*	
C9B	1.1806 (2)	0.08143 (18)	0.09064 (12)	0.0332 (5)	
H9BA	1.2307	0.1142	0.1297	0.040*	
H9BB	1.2534	0.0487	0.0611	0.040*	
C10B	1.0824 (2)	−0.00223 (16)	0.11913 (12)	0.0315 (5)	
H10C	1.0311	−0.0347	0.0803	0.038*	
H10D	1.1413	−0.0557	0.1414	0.038*	
C11B	1.0443 (2)	0.06764 (17)	0.23547 (12)	0.0322 (4)	
H11C	1.1252	0.1143	0.2262	0.039*	
H11D	1.0810	0.0067	0.2601	0.039*	

### Atomic displacement parameters (Å^2^)

**Table:**

	*U*^11^	*U*^22^	*U*^33^	*U*^12^	*U*^13^	*U*^23^
I	0.03535 (8)	0.03358 (7)	0.05708 (10)	−0.00127 (7)	−0.00778 (7)	0.01797 (7)
Fe	0.01560 (10)	0.02019 (11)	0.01862 (11)	0.00004 (10)	0.00055 (9)	0.00102 (10)
O1A	0.0264 (8)	0.0351 (7)	0.0216 (7)	−0.0061 (6)	0.0031 (5)	−0.0040 (5)
O1B	0.0174 (6)	0.0267 (6)	0.0321 (7)	0.0016 (5)	0.0029 (5)	0.0081 (5)
N1A	0.0186 (7)	0.0219 (7)	0.0203 (7)	−0.0002 (6)	0.0024 (6)	0.0010 (6)
N2A	0.0264 (8)	0.0245 (7)	0.0233 (8)	−0.0040 (6)	−0.0009 (7)	0.0012 (6)
N1B	0.0131 (7)	0.0245 (7)	0.0219 (7)	0.0000 (6)	0.0008 (6)	0.0021 (6)
N2B	0.0220 (8)	0.0217 (7)	0.0314 (9)	−0.0003 (6)	−0.0027 (7)	0.0021 (6)
C1A	0.0229 (10)	0.0284 (9)	0.0215 (8)	0.0018 (7)	−0.0041 (7)	−0.0035 (7)
C2A	0.0375 (13)	0.0475 (13)	0.0305 (11)	0.0073 (10)	−0.0024 (9)	−0.0118 (9)
C3A	0.0488 (16)	0.0396 (12)	0.0466 (13)	0.0141 (11)	−0.0102 (11)	−0.0189 (10)
C4A	0.0655 (18)	0.0248 (10)	0.0439 (14)	0.0010 (11)	−0.0180 (13)	−0.0027 (9)
C5A	0.0441 (13)	0.0296 (11)	0.0318 (11)	−0.0085 (10)	−0.0093 (9)	0.0050 (9)
C6A	0.0268 (10)	0.0275 (9)	0.0217 (9)	−0.0018 (8)	−0.0071 (7)	0.0017 (7)
C7A	0.0169 (8)	0.0316 (9)	0.0264 (10)	−0.0027 (7)	0.0000 (7)	0.0002 (7)
C8A	0.0231 (9)	0.0333 (11)	0.0314 (10)	0.0005 (8)	0.0099 (7)	−0.0013 (8)
C9A	0.0370 (12)	0.0345 (11)	0.0267 (10)	−0.0021 (9)	0.0115 (8)	−0.0040 (8)
C10A	0.0400 (13)	0.0268 (9)	0.0246 (9)	−0.0037 (8)	0.0064 (8)	−0.0060 (7)
C11A	0.0376 (12)	0.0371 (11)	0.0267 (11)	−0.0007 (9)	−0.0071 (9)	0.0059 (8)
C1B	0.0217 (10)	0.0257 (8)	0.0204 (8)	0.0031 (6)	−0.0008 (6)	0.0043 (6)
C2B	0.0251 (10)	0.0347 (10)	0.0331 (10)	0.0072 (8)	0.0044 (9)	0.0054 (9)
C3B	0.0404 (12)	0.0304 (10)	0.0350 (12)	0.0141 (9)	−0.0024 (10)	−0.0017 (9)
C4B	0.0372 (13)	0.0243 (9)	0.0373 (12)	0.0035 (9)	−0.0072 (9)	0.0026 (8)
C5B	0.0262 (10)	0.0298 (10)	0.0260 (10)	−0.0022 (8)	−0.0030 (8)	0.0049 (8)
C6B	0.0206 (9)	0.0242 (8)	0.0201 (9)	0.0007 (7)	−0.0009 (7)	0.0041 (7)
C7B	0.0211 (9)	0.0297 (10)	0.0233 (9)	0.0015 (7)	0.0028 (7)	0.0083 (7)
C8B	0.0195 (8)	0.0346 (10)	0.0315 (10)	0.0057 (9)	0.0086 (7)	0.0007 (9)
C9B	0.0188 (9)	0.0382 (11)	0.0427 (13)	0.0100 (9)	0.0023 (9)	−0.0006 (9)
C10B	0.0285 (11)	0.0273 (10)	0.0388 (12)	0.0089 (8)	−0.0002 (9)	0.0005 (8)
C11B	0.0325 (11)	0.0343 (10)	0.0296 (11)	0.0014 (8)	−0.0115 (9)	0.0047 (9)

### Geometric parameters (Å, °)

**Table:**

Fe—O1B	1.8898 (13)	C8A—H8AA	0.9900
Fe—O1A	1.8999 (14)	C8A—H8AB	0.9900
Fe—N1B	2.1805 (15)	C9A—C10A	1.517 (3)
Fe—N1A	2.2062 (15)	C9A—H9AA	0.9900
Fe—N2A	2.2062 (17)	C9A—H9AB	0.9900
Fe—N2B	2.2158 (17)	C10A—H10A	0.9900
O1A—C1A	1.332 (2)	C10A—H10B	0.9900
O1B—C1B	1.337 (2)	C11A—C11B	1.504 (3)
N1A—C7A	1.486 (2)	C11A—H11A	0.9900
N1A—C8A	1.493 (2)	C11A—H11B	0.9900
N1A—H1AA	0.9300	C1B—C2B	1.399 (3)
N2A—C10A	1.475 (3)	C1B—C6B	1.401 (3)
N2A—C11A	1.488 (3)	C2B—C3B	1.383 (3)
N2A—H2AA	0.9300	C2B—H2BB	0.9500
N1B—C7B	1.496 (2)	C3B—C4B	1.386 (3)
N1B—C8B	1.501 (2)	C3B—H3BA	0.9500
N1B—H1BA	0.9300	C4B—C5B	1.388 (3)
N2B—C10B	1.474 (3)	C4B—H4BA	0.9500
N2B—C11B	1.486 (3)	C5B—C6B	1.402 (3)
N2B—H2BA	0.9300	C5B—H5BA	0.9500
C1A—C2A	1.398 (3)	C6B—C7B	1.493 (3)
C1A—C6A	1.408 (3)	C7B—H7BA	0.9900
C2A—C3A	1.387 (3)	C7B—H7BB	0.9900
C2A—H2AB	0.9500	C8B—C9B	1.505 (3)
C3A—C4A	1.385 (4)	C8B—H8BA	0.9900
C3A—H3AA	0.9500	C8B—H8BB	0.9900
C4A—C5A	1.368 (4)	C9B—C10B	1.533 (3)
C4A—H4AA	0.9500	C9B—H9BA	0.9900
C5A—C6A	1.390 (3)	C9B—H9BB	0.9900
C5A—H5AA	0.9500	C10B—H10C	0.9900
C6A—C7A	1.497 (3)	C10B—H10D	0.9900
C7A—H7AA	0.9900	C11B—H11C	0.9900
C7A—H7AB	0.9900	C11B—H11D	0.9900
C8A—C9A	1.518 (3)		
			
O1B—Fe—O1A	99.79 (6)	C9A—C8A—H8AB	108.8
O1B—Fe—N1B	90.69 (6)	H8AA—C8A—H8AB	107.7
O1A—Fe—N1B	92.02 (6)	C10A—C9A—C8A	115.90 (17)
O1B—Fe—N1A	90.78 (6)	C10A—C9A—H9AA	108.3
O1A—Fe—N1A	90.31 (6)	C8A—C9A—H9AA	108.3
N1B—Fe—N1A	177.01 (6)	C10A—C9A—H9AB	108.3
O1B—Fe—N2A	91.25 (6)	C8A—C9A—H9AB	108.3
O1A—Fe—N2A	167.81 (6)	H9AA—C9A—H9AB	107.4
N1B—Fe—N2A	92.99 (6)	N2A—C10A—C9A	113.59 (16)
N1A—Fe—N2A	84.37 (6)	N2A—C10A—H10A	108.8
O1B—Fe—N2B	168.01 (6)	C9A—C10A—H10A	108.8
O1A—Fe—N2B	91.80 (6)	N2A—C10A—H10B	108.8
N1B—Fe—N2B	85.82 (6)	C9A—C10A—H10B	108.8
N1A—Fe—N2B	92.22 (6)	H10A—C10A—H10B	107.7
N2A—Fe—N2B	77.50 (6)	N2A—C11A—C11B	109.34 (17)
C1A—O1A—Fe	128.92 (12)	N2A—C11A—H11A	109.8
C1B—O1B—Fe	130.83 (12)	C11B—C11A—H11A	109.8
C7A—N1A—C8A	107.79 (15)	N2A—C11A—H11B	109.8
C7A—N1A—Fe	110.01 (11)	C11B—C11A—H11B	109.8
C8A—N1A—Fe	112.91 (12)	H11A—C11A—H11B	108.3
C7A—N1A—H1AA	108.7	O1B—C1B—C2B	119.97 (17)
C8A—N1A—H1AA	108.7	O1B—C1B—C6B	120.18 (16)
Fe—N1A—H1AA	108.7	C2B—C1B—C6B	119.82 (17)
C10A—N2A—C11A	112.66 (17)	C3B—C2B—C1B	119.9 (2)
C10A—N2A—Fe	116.09 (13)	C3B—C2B—H2BB	120.0
C11A—N2A—Fe	111.11 (13)	C1B—C2B—H2BB	120.0
C10A—N2A—H2AA	105.3	C2B—C3B—C4B	121.0 (2)
C11A—N2A—H2AA	105.3	C2B—C3B—H3BA	119.5
Fe—N2A—H2AA	105.3	C4B—C3B—H3BA	119.5
C7B—N1B—C8B	107.20 (15)	C3B—C4B—C5B	119.31 (19)
C7B—N1B—Fe	110.74 (11)	C3B—C4B—H4BA	120.3
C8B—N1B—Fe	112.99 (12)	C5B—C4B—H4BA	120.3
C7B—N1B—H1BA	108.6	C4B—C5B—C6B	120.9 (2)
C8B—N1B—H1BA	108.6	C4B—C5B—H5BA	119.6
Fe—N1B—H1BA	108.6	C6B—C5B—H5BA	119.6
C10B—N2B—C11B	112.46 (17)	C1B—C6B—C5B	119.06 (18)
C10B—N2B—Fe	116.31 (13)	C1B—C6B—C7B	120.53 (17)
C11B—N2B—Fe	111.58 (12)	C5B—C6B—C7B	119.96 (18)
C10B—N2B—H2BA	105.1	C6B—C7B—N1B	115.16 (15)
C11B—N2B—H2BA	105.1	C6B—C7B—H7BA	108.5
Fe—N2B—H2BA	105.1	N1B—C7B—H7BA	108.5
O1A—C1A—C2A	120.62 (19)	C6B—C7B—H7BB	108.5
O1A—C1A—C6A	120.15 (17)	N1B—C7B—H7BB	108.5
C2A—C1A—C6A	119.18 (19)	H7BA—C7B—H7BB	107.5
C3A—C2A—C1A	119.8 (2)	N1B—C8B—C9B	114.22 (16)
C3A—C2A—H2AB	120.1	N1B—C8B—H8BA	108.7
C1A—C2A—H2AB	120.1	C9B—C8B—H8BA	108.7
C4A—C3A—C2A	120.9 (2)	N1B—C8B—H8BB	108.7
C4A—C3A—H3AA	119.5	C9B—C8B—H8BB	108.7
C2A—C3A—H3AA	119.5	H8BA—C8B—H8BB	107.6
C5A—C4A—C3A	119.3 (2)	C8B—C9B—C10B	115.07 (18)
C5A—C4A—H4AA	120.3	C8B—C9B—H9BA	108.5
C3A—C4A—H4AA	120.3	C10B—C9B—H9BA	108.5
C4A—C5A—C6A	121.5 (2)	C8B—C9B—H9BB	108.5
C4A—C5A—H5AA	119.2	C10B—C9B—H9BB	108.5
C6A—C5A—H5AA	119.2	H9BA—C9B—H9BB	107.5
C5A—C6A—C1A	119.21 (19)	N2B—C10B—C9B	113.66 (17)
C5A—C6A—C7A	121.14 (19)	N2B—C10B—H10C	108.8
C1A—C6A—C7A	119.29 (17)	C9B—C10B—H10C	108.8
N1A—C7A—C6A	115.14 (16)	N2B—C10B—H10D	108.8
N1A—C7A—H7AA	108.5	C9B—C10B—H10D	108.8
C6A—C7A—H7AA	108.5	H10C—C10B—H10D	107.7
N1A—C7A—H7AB	108.5	N2B—C11B—C11A	109.10 (17)
C6A—C7A—H7AB	108.5	N2B—C11B—H11C	109.9
H7AA—C7A—H7AB	107.5	C11A—C11B—H11C	109.9
N1A—C8A—C9A	113.79 (17)	N2B—C11B—H11D	109.9
N1A—C8A—H8AA	108.8	C11A—C11B—H11D	109.9
C9A—C8A—H8AA	108.8	H11C—C11B—H11D	108.3
N1A—C8A—H8AB	108.8		
			
O1B—Fe—O1A—C1A	−121.63 (16)	C6A—C1A—C2A—C3A	−1.0 (3)
N1B—Fe—O1A—C1A	147.32 (16)	C1A—C2A—C3A—C4A	0.3 (4)
N1A—Fe—O1A—C1A	−30.79 (16)	C2A—C3A—C4A—C5A	−0.2 (4)
N2A—Fe—O1A—C1A	33.1 (4)	C3A—C4A—C5A—C6A	0.8 (4)
N2B—Fe—O1A—C1A	61.45 (16)	C4A—C5A—C6A—C1A	−1.5 (3)
O1A—Fe—O1B—C1B	−116.70 (15)	C4A—C5A—C6A—C7A	171.5 (2)
N1B—Fe—O1B—C1B	−24.54 (15)	O1A—C1A—C6A—C5A	178.97 (18)
N1A—Fe—O1B—C1B	152.85 (15)	C2A—C1A—C6A—C5A	1.6 (3)
N2A—Fe—O1B—C1B	68.47 (16)	O1A—C1A—C6A—C7A	5.8 (3)
N2B—Fe—O1B—C1B	48.3 (4)	C2A—C1A—C6A—C7A	−171.55 (19)
O1B—Fe—N1A—C7A	82.12 (12)	C8A—N1A—C7A—C6A	−178.68 (16)
O1A—Fe—N1A—C7A	−17.68 (12)	Fe—N1A—C7A—C6A	57.82 (18)
N2A—Fe—N1A—C7A	173.30 (13)	C5A—C6A—C7A—N1A	127.69 (19)
N2B—Fe—N1A—C7A	−109.49 (12)	C1A—C6A—C7A—N1A	−59.3 (2)
O1B—Fe—N1A—C8A	−38.34 (13)	C7A—N1A—C8A—C9A	170.82 (17)
O1A—Fe—N1A—C8A	−138.14 (13)	Fe—N1A—C8A—C9A	−67.46 (19)
N2A—Fe—N1A—C8A	52.85 (13)	N1A—C8A—C9A—C10A	66.2 (2)
N2B—Fe—N1A—C8A	130.06 (13)	C11A—N2A—C10A—C9A	−66.6 (2)
O1B—Fe—N2A—C10A	38.47 (13)	Fe—N2A—C10A—C9A	63.1 (2)
O1A—Fe—N2A—C10A	−116.6 (3)	C8A—C9A—C10A—N2A	−62.7 (3)
N1B—Fe—N2A—C10A	129.23 (13)	C10A—N2A—C11A—C11B	172.73 (16)
N1A—Fe—N2A—C10A	−52.18 (13)	Fe—N2A—C11A—C11B	40.5 (2)
N2B—Fe—N2A—C10A	−145.72 (14)	Fe—O1B—C1B—C2B	−146.23 (16)
O1B—Fe—N2A—C11A	168.93 (14)	Fe—O1B—C1B—C6B	35.7 (2)
O1A—Fe—N2A—C11A	13.8 (4)	O1B—C1B—C2B—C3B	−176.72 (19)
N1B—Fe—N2A—C11A	−100.31 (14)	C6B—C1B—C2B—C3B	1.3 (3)
N1A—Fe—N2A—C11A	78.27 (14)	C1B—C2B—C3B—C4B	−2.0 (3)
N2B—Fe—N2A—C11A	−15.27 (14)	C2B—C3B—C4B—C5B	0.6 (3)
O1B—Fe—N1B—C7B	−20.81 (13)	C3B—C4B—C5B—C6B	1.6 (3)
O1A—Fe—N1B—C7B	79.02 (13)	O1B—C1B—C6B—C5B	178.81 (17)
N2A—Fe—N1B—C7B	−112.10 (12)	C2B—C1B—C6B—C5B	0.8 (3)
N2B—Fe—N1B—C7B	170.67 (13)	O1B—C1B—C6B—C7B	6.5 (3)
O1B—Fe—N1B—C8B	−141.09 (13)	C2B—C1B—C6B—C7B	−171.54 (18)
O1A—Fe—N1B—C8B	−41.26 (13)	C4B—C5B—C6B—C1B	−2.2 (3)
N2A—Fe—N1B—C8B	127.62 (12)	C4B—C5B—C6B—C7B	170.12 (19)
N2B—Fe—N1B—C8B	50.39 (13)	C1B—C6B—C7B—N1B	−56.4 (2)
O1B—Fe—N2B—C10B	−122.7 (3)	C5B—C6B—C7B—N1B	131.38 (19)
O1A—Fe—N2B—C10B	42.58 (14)	C8B—N1B—C7B—C6B	−178.95 (16)
N1B—Fe—N2B—C10B	−49.31 (14)	Fe—N1B—C7B—C6B	57.38 (19)
N1A—Fe—N2B—C10B	132.95 (14)	C7B—N1B—C8B—C9B	170.13 (18)
N2A—Fe—N2B—C10B	−143.31 (15)	Fe—N1B—C8B—C9B	−67.59 (19)
O1B—Fe—N2B—C11B	8.1 (4)	N1B—C8B—C9B—C10B	68.4 (2)
O1A—Fe—N2B—C11B	173.41 (14)	C11B—N2B—C10B—C9B	−69.6 (2)
N1B—Fe—N2B—C11B	81.52 (14)	Fe—N2B—C10B—C9B	60.8 (2)
N1A—Fe—N2B—C11B	−96.21 (13)	C8B—C9B—C10B—N2B	−63.9 (3)
N2A—Fe—N2B—C11B	−12.48 (13)	C10B—N2B—C11B—C11A	170.70 (16)
Fe—O1A—C1A—C2A	−141.17 (18)	Fe—N2B—C11B—C11A	37.91 (19)
Fe—O1A—C1A—C6A	41.5 (2)	N2A—C11A—C11B—N2B	−51.6 (2)
O1A—C1A—C2A—C3A	−178.4 (2)		

### Hydrogen-bond geometry (Å, °)

**Table:**

*D*—H···*A*	*D*—H	H···*A*	*D*···*A*	*D*—H···*A*
N1A—H1AA···I	0.93	2.77	3.6800 (16)	168
N2A—H2AA···I^i^	0.93	2.96	3.8227 (17)	155
N1B—H1BA···I^i^	0.93	2.80	3.7285 (16)	178
N2B—H2BA···I	0.93	2.81	3.6911 (17)	158
C11B—H11C···I^i^	0.99	3.10	3.942 (2)	144

Symmetry codes: (i) −*x*+2, *y*+1/2, −*z*+1/2.
